# A Critical Assessment of Antenatal Monitoring for Fetal Well-Being in Down Syndrome Pregnancies

**DOI:** 10.3390/diagnostics16010039

**Published:** 2025-12-22

**Authors:** Juliet C. Bishop, Angie C. Jelin, Ahizechukwu C. Eke, Christine B. Hertenstein, Amanda Jones, Clark T. Johnson, Karin Blakemore

**Affiliations:** Division of Maternal-Fetal Medicine, Department of Gynecology & Obstetrics, The Johns Hopkins University School of Medicine, Baltimore, MD 21205, USA

**Keywords:** Down syndrome, trisomy 21, antenatal fetal surveillance, fetal well-being, fetal demise, fetal growth restriction, umbilical artery Doppler, nonstress testing, polyhydramnios, fetal anomalies

## Abstract

**Background/Objectives:** The antenatal management of Down syndrome (DS) is difficult as it is associated with a high risk for in utero fetal demise (IUFD) with a paucity of literature to guide antenatal surveillance. Avoidance of preterm delivery in the DS fetus, so commonly affected by anomalies, compounds the dichotomy of achieving term delivery while balancing against the risk for IUFD as gestational age advances. Higher-performing tests are needed to predict, and, thus, hopefully prevent, both preterm delivery and fetal mortality. Our study was undertaken to evaluate the performance metrics of current antenatal surveillance parameters that might suggest an increased risk for IUFD. **Methods:** We studied a retrospective cohort of all continuing pregnancies with a cytogenetically confirmed DS fetus between 2009 and 2019 at a single institution. Cases were investigated for abnormalities in fetal growth, anatomy, UA Doppler, and amniotic fluid volume to analyze their interrelationships and their association with the primary outcome, IUFD. Nonstress testing (NST) and biophysical profile data as available were also reviewed for analysis on each case. Maternal demographic data were also collected. **Results:** A total of 41 DS pregnancies at >20 weeks gestation were included. Eight (19.5%) resulted in IUFD, while thirty-three (80.5%) resulted in live birth. Between these groups, there was no significant difference in the incidence of fetal structural anomalies. FGR was present in 8/41 fetuses or 19.5% of all cases. FGR was present in 1 of 8 (12.5%) IUFD cases and 7 of 33 (21.2%) live births (*p* = 0.50). Thus, notably, 87.5% (7/8) of the IUFDs occurred in the absence of FGR. Furthermore, 1/8 (12.5%) FGR cases resulted in IUFD vs. 7/33 (21.2%) of non-FGR cases (*p* = 0.50). In DS fetuses after 24 weeks gestation, UA Doppler abnormalities developed in 75% of FGR cases (6/8) and in 64.5% of normally grown cases (20/31) (*p* = 0.33). Abnormal UA Dopplers were noted in 83.3% of IUFD and in 84.8% of liveborn cases (*p* = 0.34). Eleven of thirty-three live births, however, underwent iatrogenic delivery secondary to worsening fetal surveillance, including ten with worsening UA Doppler indices. There was an increased frequency of abnormal NST in the IUFD group (66.7% vs. 23.8%), although this difference did not reach statistical significance. Polyhydramnios was more frequent in the IUFD group (62.5% vs. 16.1%, *p* = 0.04). **Conclusions:** Aside from polyhydramnios, no fetal surveillance parameter demonstrated an association with IUFD that reached statistical significance. A majority of fetuses with DS are normally grown and demonstrate abnormal UA Doppler indices in the absence of FGR. Within our cohort, a substantial number of liveborn deliveries were prompted following worsening UA Dopplers. Both polyhydramnios and UA Doppler indices are worthy of further investigation to inform clinically useful fetal surveillance strategies in DS.

## 1. Introduction

Down syndrome (DS) is the most common liveborn chromosomal aneuploidy with a prevalence of 1.4 per 1000 births in the United States [1,2]. The prevalence of DS live births is also dependent on the availability and uptake of prenatal diagnosis and of pregnancy termination [3,4]. The increased risk of in utero fetal demise (IUFD) is well-recognized, with the rate of demise in DS-affected pregnancies above 20 weeks gestation reported to be 5–33% [5,6,7,8,9,10].

For liveborns with DS, it has been found that gestational age at delivery, birthweight, and presence of structural anomalies are predictors of postnatal survival [8]. There is, however, limited information on the natural history of DS prenatally, and even less information to help guide antenatal surveillance for these high-risk pregnancies. Avoidance of preterm delivery of the DS fetus, so commonly affected by anomalies, compounds the dichotomy of achieving a term delivery while balancing against the risk for IUFD as gestation advances [6]. For patients who continue a pregnancy affected by DS, appropriate counseling to address concerns of prognosis and outcome is challenging as there is no uniform clinical guidance for antenatal fetal surveillance.

Placental insufficiency is associated with perinatal mortality risk often presenting as fetal growth restriction (FGR) [11,12]. Accordingly, FGR is a routine indication for antenatal surveillance using nonstress testing (NST) and fetal Doppler assessment [13,14]. Previous studies have found that 72–83 percent of DS stillbirths are not growth-restricted [6,7,9]. Thus, restricting routine fetal surveillance using this indication may not serve to identify DS fetuses at highest risk for IUFD. In continuing DS pregnancies, as in general, Doppler assessment of the umbilical artery (UA) can be employed for pregnancies at risk for placental insufficiency, while NST and biophysical profile (BPP) are the methods most used to assess fetal well-being for a variety of maternal–fetal indications.

Our study was undertaken to investigate continuing DS pregnancies for antenatal surveillance parameters that might suggest an increased risk for IUFD. We evaluated the performance metrics of current antenatal surveillance tests such as NST and BPPs, as well as other fetal surveillance parameters such as fetal growth, UA Dopplers, fetal phenotype with respect to anomalies, and amniotic fluid volume. We further examined the relationship between FGR and abnormal UA Doppler indices in our study population.

The study was confined to a time period during which a specific protocol for antenatal surveillance for fetal DS was not in effect to better gain insight into the condition’s natural history.

## 2. Materials and Methods

We studied a retrospective cohort of all continuing pregnancies >20 weeks gestation with a cytogenetically confirmed DS fetus between 2009 and 2019 at our institution, which serves the general population through routine anomaly screening and is a referral center for fetal anomalies. Patients were identified from our perinatal ultrasound database. Indications for cytogenetic analysis included an abnormal cell-free DNA screen, maternal age, and ultrasound abnormalities. Excluded were patients electing for pregnancy termination, not continuing care at our center, or whose follow-up antepartum and outcome data were unknown. Diagnostic testing and aneuploidy screening results pertinent to the diagnosis of trisomy 21 were obtained from our Department of Pathology, Cytogenetics Laboratory, and/or commercial testing labs. Medical records were interrogated for abnormalities in growth, anatomy, UA Doppler assessments, amniotic fluid index (AFI), NST and BPP data as available, and perinatal outcomes. A standardized antenatal surveillance protocol specific to DS continuing pregnancy was not in use during the study period other than serial fetal growth in combination with UA Doppler assessments, usually monthly, in the third trimester. Rather, standard indications for NST and AFI were employed. The use of BPP was limited to fetuses with an abnormal NST defined as non-reactive and/or non-reassuring with FHR decelerations.

All prenatal sonograms were performed by ultrasonographers with Maternal–Fetal Medicine specialists using Voluson E7, E8, or E10 GE Healthcare ultrasound machines. A detailed ultrasound anatomic evaluation was performed after 18 weeks regardless of gestational age at presentation to our center. Ultrasound, antepartum surveillance measures, delivery indications, and perinatal outcome data were extracted from patient medical records. Our primary outcome was IUFD in fetuses diagnosed with DS. Secondary outcomes included FGR, fetal structural anomalies, antenatal fetal testing abnormalities including UA Doppler assessments, NST and BPPs, amniotic fluid abnormalities, gestational age at delivery, birth weight, and neonatal intensive care unit (NICU) stay.

For data analysis, categorical variables are reported as proportions, and continuous measures are reported as means, as described in Table 1 and Table 2. Differences between groups were assessed with Fisher’s exact tests and Chi-square tests (where applicable) for statistical analysis of categorical variables. Student’s *t*-test was used for continuous variables. All statistical analysis was conducted in Stata, version 15.1 (StataCorp, College Station, TX, USA, 2019), with a *p* value of <0.05 being statistically significant.

The study protocol was reviewed and approved by the Johns Hopkins University Institutional Review Board (IRB#: 00095781).

## 3. Results

During the study period, there were 41 continuing DS-affected pregnancies >20 weeks gestation which met the inclusion criteria. All cases were singletons. Eight (19.5%) resulted in IUFD, while thirty-three (79.5%) resulted in live birth. Between these groups, there was no significant difference in maternal or gestational demographics (Table 1), nor in the incidence of fetal structural anomalies (Table 2).

Approximately half of the women (22 of 41; 53%) were of advanced maternal age (Table 1). Approximately half were White (51.2%), one third were African American (34.1%), 12.2% were of Hispanic descent, and 2.4% were of Asian descent. The majority were either married (68%) or had a partner (7%). Most (87.8%) denied drug, alcohol, or tobacco use during pregnancy. Approximately half of the cohort was healthy without notable maternal comorbidity. Maternal comorbidities were various, with no one disease exceedingly present amongst the cohort as a whole. Most were multiparous (70%); 30% were nulliparous.

FGR was present in 7 of 33 (21.2%) liveborn cases and in 1 of 8 (12.5%) IUFD cases (*p* = 0.50). Moreover, the vast majority of IUFD cases (87.5%) occurred in normally grown fetuses. Lastly, only 1 of 8 FGR fetuses had IUFD (12.5%), while 7 of 33 (21.2%) normally grown fetuses had IUFD (*p* = 0.50). No statistically significant association between FGR and IUFD was detected in DS pregnancies. Fetal structural anomalies consisted of those commonly associated with DS. Brain abnormalities included cerebral ventriculomegaly, hypoplastic cerebellum, vermian hypoplasia, and Dandy Walker malformation. Cardiac anomalies included atrioventricular canal defects (AVCDs), other septal defects, double outlet right ventricle, pulmonary atresia, and one complex cardiac defect with AVCD, aortic coarctation, and a cleft mitral valve. Renal abnormalities included urinary tract dilations, renal cysts, renal artery duplication, and echogenic kidneys. Gastrointestinal abnormalities included duodenal atresia, small stomach, isolated enlarged stomach, isolated dilated bowel, and echogenic liver. Limb findings included fifth finger clinodactyly, clubbed feet, sandal gap toes, and short femur and/or humerus. Neither the presence of any major structural anomaly/soft markers nor the presence of any particular subcategory of fetal anomaly demonstrated a statistically significant difference between the IUFD and liveborn groups (for any major anomaly, OR 1.56 [95% [CI 0.33, 7.21, *p* = 0.551]).

The mean gestational age of the eight IUFD cases was 31+4/7 weeks. Third-trimester UA Doppler velocimetry was assessed in all but two peri-viable IUFD cases (at 21 weeks and 24 weeks; neither had FGR). All other (*n* = 39) cases were delivered at >28 weeks and underwent UA Doppler assessments. In our analysis we found that 75% of fetuses (6/8) in the FGR subset versus 64.5% of fetuses (20/31) in the normally grown subset demonstrated UA Doppler abnormalities (OR 0.90 [95% CI 0.31, 2.57, *p* = 0.334]). Abnormal UA Dopplers were noted in 83.3% of IUFD and in 84.8% of liveborn cases despite FGR being present in only 12.5% and 21.2% of each group, respectively (Table 2). Thus, there was no statistically significant association between abnormal UA Doppler and IUFD in the DS fetuses. Although there was an increased frequency of abnormal NST in the IUFD group (66.7% vs. 21.7%), this difference also did not reach statistical significance. Polyhydramnios was more frequent in the IUFD group (62.5% vs. 18.2%, *p* = 0.040).

Of the 33 live births, 11 cases (5 normally grown, 6 FGR) underwent iatrogenic delivery secondary to worsening fetal surveillance parameters. In all 11 cases, elevated UA Dopplers were detected at a follow-up growth ultrasound at a gestational age preceding the EGA of delivery. In 10 of 11 cases, subsequent antenatal fetal surveillance detected worsening UA Dopplers for which extended monitoring or additional fetal testing was performed and demonstrated non-reassuring fetal status leading to delivery. Delivery indications included NRFHTs on extended fetal monitoring, a worsening biophysical profile, the development of REDF, or fetal hydrops. In 1 of 11 cases, which was normally grown, induction of labor was performed upon reaching term gestational age with elevated UA Dopplers and a 6/8 BPP. No cases of preterm delivery were for an isolated finding of abnormal UA Doppler indices. The mean gestational age at delivery for these 11 cases was 35 weeks, while that of the remaining 22 cases delivered for routine indications, inclusive of spontaneous labor, was 37+4/7 weeks. Amongst the live births, no cord gases showed significant acidemia: mean UA pH 7.26 (range 7.17–7.36).

## 4. Discussion

### 4.1. Principal Findings

In the current study, a majority of fetuses with DS were normally grown, and a majority demonstrated abnormal UA Doppler indices in the absence of FGR. Our data did not demonstrate FGR or abnormal UA Doppler indices to be useful markers for IUFD in DS. No statistically significant association was detected within this limited sample.

While our data were unable to discriminate whether surveillance by NST or BPP could be used as a predictive test for demise in DS, in one third of non-IUFD cases, the use of these measures along with UA Doppler indices did reveal concerns regarding fetal well-being, prompting increased monitoring or delivery resulting in live birth.

### 4.2. Results

Antepartum fetal surveillance techniques are used to assess fetal well-being with the goal of preventing IUFD in pregnancies at higher risk [15,16]. Fetal surveillance is routinely used for maternal conditions such as hypertension and pregestational diabetes, as well as for conditions such as FGR, oligohydramnios, or fetal structural abnormalities [16,17,18]. Though pregnancies affected by DS are known to be at higher risk for IUFD [6], DS is not a clearly recognized indication for antenatal fetal surveillance. What fetal characteristics, if any, would place a DS fetus at higher risk for demise and warrant antenatal fetal surveillance is also unclear.

Our DS cohort’s 19.5% rate of IUFD is within the range (7–33%) previously reported [5,6,7,9,10]. A few studies have suggested fetal characteristics associated with higher risks of antepartum and postnatal demise in DS, including FGR and atypical cardiac anomalies [9,10,19]. Yao et al. found that over one third of DS stillbirths were growth-restricted; these authors suggested that routine surveillance of fetal growth and routine indications for antenatal fetal surveillance may reduce the risk of perinatal mortality in DS pregnancies [9]. Their study, as well as an earlier study by Guseh et al. [7], also raised the point, however, that routine surveillance strategies may not be effective for the two thirds of DS stillbirth cases that are not affected by growth restriction. Within our cohort, there was no significant difference in FGR or the presence of anomalies—cardiac or any other—when comparing live births to IUFDs. The cohort had 21% of live births versus 12.5% of IUFDs being growth-restricted, with similar estimated fetal weight percentile means and ranges between the groups. The vast majority (87.5%) of IUFD cases were not growth-restricted, demonstrating FGR’s limited utility as an indicator for increased fetal surveillance in trisomy 21. Our findings endorse the notion that the majority of DS demises are not mediated through FGR.

Oligohydramnios and polyhydramnios are also indications for initiating antenatal fetal surveillance [17,20,21,22]. Within our cohort, there was no significant difference in oligohydramnios in the live birth group compared to the IUFD group. Interestingly, polyhydramnios was more frequent in the IUFD group and was the only measure that reached statistical significance. Polyhydramnios may result from a structural abnormality such as duodenal atresia or from neurologic dysfunction related to aneuploidy. The increased frequency of polyhydramnios in DS IUFDs suggests that it may reflect a greater degree of dysfunction in DS pregnancies. Future studies are needed to confirm whether polyhydramnios elevates the risk of fetal demise in DS.

In investigating antenatal fetal surveillance strategies in DS pregnancies, we evaluated UA Doppler velocimetry, NST, and BPPs. Currently, there is no evidence that UA Doppler velocimetry provides information about fetal well-being in a fetus with normal growth, and, thus, it is not routinely measured in the normally grown fetus [23]. Two previous studies found that up to 50% of DS fetuses had abnormal UA Doppler indices in the late second or third trimester with no correlation to the presence of major structural anomaly or evidence of placental insufficiency [24,25]. A slightly later study, however, determined that 53% of DS fetuses delivered for reasons of “non-reassuring fetal surveillance” had histopathologic findings of placental insufficiency as evidenced by significant infarcts or subchorionic fibrin deposition [7]. Within our cohort, abnormal UA Doppler indices were noted in a majority of all DS pregnancies, both liveborn and IUFDs, despite FGR being present in only a minority in each group.

No statistically significant association was detected within our limited sample. However, our data support that abnormal UA Doppler indices are a very common albeit physiologically unexplained attribute of DS fetuses, including those that are normally grown.

During the study period, abnormalities in fetal surveillance, when encountered, as per usual practice for high-risk pregnancies, were similarly acted upon in our DS cohort, occurring in one third (11/33) of liveborn cases. It is important to recognize that 5 of 33 (15%) live births in our cohort resulted following iatrogenic delivery secondary to worsening UA Doppler indices in normally grown fetuses who otherwise would not have been monitored under routine surveillance indications. Our study was not powered for correlation of variables; however, there was no apparent notable trend between abnormal UA Doppler indices and the presence of major fetal structural anomaly. Although there was an increased frequency of abnormal NST in the IUFD group (66.7% vs. 21.7%), this difference did not reach statistical significance. With few BPPs performed in the cohort overall, there was no difference between groups that was statistically significant. Our findings suggest that there may be fundamental enigmatic physiological differences in DS fetuses and/or their placentas underlying their demonstrated altered antenatal surveillance patterns.

### 4.3. Clinical Implications

Current antepartum surveillance protocols which employ FGR as the indication for initiating fetal surveillance are inadequate to identify DS fetuses at risk for impending IUFD. FGR serves as one of the few fetal conditions to represent placental insufficiency for which to initiate antenatal surveillance. Perhaps in recognizing that the chromosomally abnormal fetus necessitates a chromosomally abnormal placenta, one in which there is a known elevated risk of perinatal mortality, then consideration could be made to include the use of antenatal surveillance tests, including UA Doppler indices, in the monitoring of DS pregnancies. Heightened fetal surveillance of the DS fetus during the third trimester to identify worsening of UA Doppler indices along with NST abnormalities and amniotic fluid alterations may assist in delivery timing decisions to obviate an IUFD.

### 4.4. Research Implications

Our results call for further systematic study of various fetal surveillance methods in continuing pregnancies with DS. A prospective study using a standardized, comprehensive fetal surveillance protocol and larger sample size is needed to better elucidate predictors of IUFD in DS. The enigma of the extraordinarily high rate of abnormal UA Doppler indices in DS during the third trimester also warrants a larger prospective study in order to elicit the natural history and the physiological basis of this phenomenon.

### 4.5. Strengths and Limitations

This study’s strengths include a relatively large sample size for the population of interest. DS is rare (1.4 per 1000 U.S. births), and continuing pregnancies prenatally recognized as having DS are even more rare. Thus, for the study period, the number of cases was considerable. Antepartum surveillance and deliveries having been conducted at a single high-risk obstetrical referral center allowed for complete ascertainment of available data and detailed review of what circumstances and indications led to delivery. The study period, moreover, was confined to an era during which only routine indications for antepartum surveillance were employed.

Our study nonetheless had some limitations secondary to sample size. First, we were underpowered for sub-group analyses in our study. Studies with low statistical power have a low probability of detecting a true effect (if one exists), so it is possible we did not detect some pertinent associations between DS sub-groups and our primary and secondary outcomes. Second, we did not examine the association between DS and IUFD using multivariable logistic regression analyses and, therefore, could not efficiently control for confounding in our analyses. Regression analyses define models that best predict the probability of an outcome as a function of explanatory covariates [26]. It was not feasible, however, to perform multivariable logistic regression analysis in this study as small sample sizes can affect the properties of maximum likelihood estimation and bias results [27,28]. Lastly, because the study was undertaken to retrospectively research antepartum surveillance modalities that might be predictive of IUFD in pregnancies intended to continue with fetal DS during a study period in which there were no known discerning modalities due to a paucity of literature on this subject, a standard formal protocol for prospectively following all such pregnancies was not in place at the time. As a consequence, data points for fetal surveillance were limited to those derived from standard indications.

## 5. Conclusions

A majority of fetuses with DS were normally grown and demonstrated abnormal UA Doppler indices in the absence of FGR. Aside from polyhydramnios, there were no other antenatal fetal surveillance parameters that demonstrated a statistically significant association with IUFD. However, our data trends provide insight into the inherent difference in physiologic responses between a DS fetus and a chromosomally normal fetus. Certainly, using FGR as the sole indicator for implementing fetal surveillance will not address the vast majority of IUFDs which occur quite frequently in DS. Although no statistically significant association was demonstrable for NST, BPPs, or UA Dopplers with IUFD, their use in antenatal management may prompt increased fetal surveillance or delivery resulting in live birth; these parameters are worthy of further investigation. Given its greater frequency in DS IUFD, polyhydramnios should trigger closer fetal surveillance in continuing DS pregnancies. The prevention of IUFD in the DS fetus has not been attained using current fetal surveillance protocols and is worthy of future investigation.

## Figures and Tables

**Table 1:** Maternal demographic characteristics.

Maternal Characteristic	Non-IUFD (Live Birth) *n* = 33 (%)	IUFD *n* = 8 (%)	*p* Value
Maternal age			
Age > 35 y	19 (57.6)	3 (37.5)	0.436
Race			0.246
Caucasian	19 (57.6)	2 (25)	
African American	9 (27.3)	5 (62.5)	
Hispanic	4 (12.1)	1 (12.5)	
Asian	1 (3.0)	0 (0)	
Marital Status			0.066
Single	6 (16.2)	4 (50)	
Significant Other	2 (6.0)	1 (12.5)	
Married	25 (75.8)	3 (37.5)	
Maternal T/E/D use	4 (12.1)	1 (12.5)	0.683
Maternal BMI > 30	11 (33.3)	4 (50.0)	0.273
Maternal Comorbidities			0.942
None	17 (51.5)	5 (62.5)	
Diabetes	3 (9.1)	0 (0)	
Thyroid disease	3 (9.1)	0 (0)	
Coagulopathy	2 (6.0)	0 (0)	
Psychiatric	4 (12.1)	2 (25)	
GI disease	1 (3.0)	0 (0)	
Multiple comorbidities	3 (9.1)	1 (12.5)	
Parity			0.569
Nulliparous	10 (30.3)	2 (25)	
Multiparous	23 (69.7)	6 (75)	

IUFD = intrauterine fetal demise; T/E/D = tobacco, ethanol, illicit drugs; BMI = Body Mass Index; GI = gastrointestinal.

**Table 2:** Fetal and antenatal surveillance parameters.

	Non-IUFD (Live Birth) *n* = 33 (%)	IUFD *n* = 8 (%)	*p* Value
Fetal growth restriction	7 (21.2)	1 (12.5)	0.503
AGA	26 (78.8)	7 (87.5)	
EFW percentile mean, % EFW percentile range, %	29.8 <3 to 79	33.8 <1 to 75	
Fetal structural abnormality	27 (81.8)	7 (87.5)	
Brain	11 (33.3)	2 (25)	0.501
Cardiac	20 (60.6)	5 (62.5)	0.626
Renal	8 (24.4)	1 (12.5)	0.427
Gastrointestinal	10 (30.3)	3 (37.5)	0.499
Limb/musculoskeletal	17 (51.5)	3 (37.5)	0.377
Placental/cord	1 (3.0)	1 (12.5)	0.356
AFI normal	22 (66.7)	3 (37.5)	0.047
Oligohydramnios	5 (15.1)	0 (0)	0.578
Polyhydramnios	6 (18.2)	5 (62.5)	0.040
Fetal sex			
Female	19 (57.6)	3 (42.9)	0.383
Antenatal UA Doppler abnormality *Excluding 2 peri-viable cases* *	*28/33 (84.8)*	*5/6 (83.3)*	0.337 *0.661*
Normal	5 (15.1)	1 (16.7)	0.430
Elevated	26 (78.8)	4 (66.7)	0.430
AEDF	1 (3.0)	0 (0)	0.430
REDF	1 (3.0)	1 (16.7)	0.430
NST abnormality	5/23 (21.7)	4/6 (66.7)	0.056
BPP abnormality	5/21 (23.8)	0/5 (0)	0.309
Gestational age at time of delivery, weeks			0.005
<28	0 (0)	2 (25)	
28–34	6 (18.2)	2 (25)	
34–37	6 (18.2)	3 (37.5)	
>37	21 (63.6)	1 (12.5)	
Birth weight range, g	940–3775	400–2880	
NICU stay, avg. days	33 (range 1–117)	-	
	**Iatrogenic Delivery** ***n*** ** = 11 (33%)**		
Umbilical arterial pH, mean	7.26 (range 7.17–7.36)	-	

* One peri-viable case was growth-restricted; the other was not. EFW = estimated fetal weight; AFI = amniotic fluid index; UA = umbilical artery; AEDF = absent end-diastolic flow; REDF = reverse end-diastolic flow; NST = nonstress testing; BPP = biophysical profile; NICU = neonatal intensive care unit.

## Data Availability

The original contributions presented in this study are included in the article. Further inquiries can be directed to the corresponding author.
